# 2-(1*H*-Benzotriazol-1-yl)-1-(furan-2-yl)ethanol

**DOI:** 10.1107/S1600536811051798

**Published:** 2011-12-10

**Authors:** Özden Özel Güven, Meral Bayraktar, Simon J. Coles, Tuncer Hökelek

**Affiliations:** a Department of Chemistry, Zonguldak Karaelmas University, 67100 Zonguldak, Turkey; b Department of Chemistry, Southampton University, Southampton SO17 1BJ, England; c Department of Physics, Hacettepe University, 06800 Beytepe, Ankara, Turkey

## Abstract

In the title compound, C_12_H_11_N_3_O_2_, the benzotriazole ring system is approximately planar [maximum deviation = 0.008 (1) Å] and its mean plane is oriented at a dihedral angle of 24.05 (4)° with respect to the furan ring. In the crystal, O—H⋯N hydrogen bonds link the mol­ecules into chains along the *ac* diagonal. π–π stacking between the furan rings, between the triazole and benzene rings, and between the benzene rings [centroid–centroid distances = 3.724 (1), 3.786 (1) and 3.8623 (9) Å] are also observed.

## Related literature

For general background to the biological activity of benzotriazole derivatives, see: Hirokawa *et al.* (1998 ▶); Yu *et al.* (2003 ▶); Kopanska *et al.* (2004 ▶). For related structures, see: Caira *et al.* (2004 ▶); Katritzky *et al.* (2001 ▶); Özel Güven *et al.* (2008 ▶, 2010 ▶, 2011 ▶); Nanjunda Swamy *et al.* (2006 ▶).
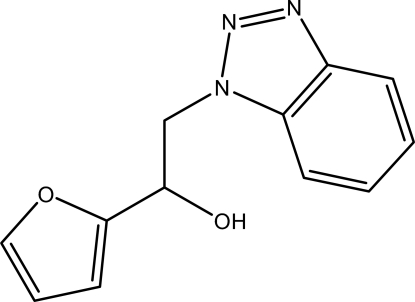


## Experimental

### 

#### Crystal data

C_12_H_11_N_3_O_2_
*M* *_r_* = 229.24
Monoclinic, 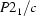

*a* = 11.3606 (4) Å
*b* = 11.1034 (4) Å
*c* = 8.7860 (2) Å
β = 96.938 (2)°
*V* = 1100.16 (6) Å^3^
*Z* = 4
Mo *K*α radiation
μ = 0.10 mm^−1^
*T* = 120 K
0.50 × 0.50 × 0.20 mm

#### Data collection

Bruker–Nonius KappaCCD diffractometer
Absorption correction: multi-scan (*SADABS*; Sheldrick, 2007 ▶) *T*_min_ = 0.953, *T*_max_ = 0.981
12372 measured reflections
2531 independent reflections
2166 reflections with *I* > 2σ(*I*)
*R*_int_ = 0.037

#### Refinement

*R*[*F* ^2^ > 2σ(*F* ^2^)] = 0.054
*wR*(*F* ^2^) = 0.139
*S* = 1.11
2531 reflections
155 parameters
H-atom parameters constrained
Δρ_max_ = 0.58 e Å^−3^
Δρ_min_ = −0.55 e Å^−3^

### 

Data collection: *COLLECT* (Nonius, 1998 ▶); cell refinement: *DENZO* (Otwinowski & Minor, 1997 ▶) and *COLLECT*; data reduction: *DENZO* and *COLLECT*; program(s) used to solve structure: *SHELXS97* (Sheldrick, 2008 ▶); program(s) used to refine structure: *SHELXL97* (Sheldrick, 2008 ▶); molecular graphics: *ORTEP-3 for Windows* (Farrugia, 1997 ▶); software used to prepare material for publication: *WinGX* (Farrugia, 1999 ▶) and *PLATON* (Spek, 2009 ▶).

## Supplementary Material

Crystal structure: contains datablock(s) I, global. DOI: 10.1107/S1600536811051798/xu5402sup1.cif

Structure factors: contains datablock(s) I. DOI: 10.1107/S1600536811051798/xu5402Isup2.hkl

Supplementary material file. DOI: 10.1107/S1600536811051798/xu5402Isup3.cml

Additional supplementary materials:  crystallographic information; 3D view; checkCIF report

## Figures and Tables

**Table 1:** Hydrogen-bond geometry (Å, °)

*D*—H⋯*A*	*D*—H	H⋯*A*	*D*⋯*A*	*D*—H⋯*A*
O1—H1⋯N3^i^	0.82	2.26	2.7968 (18)	123

Symmetry code: (i) 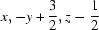.

**Fig. 1.**
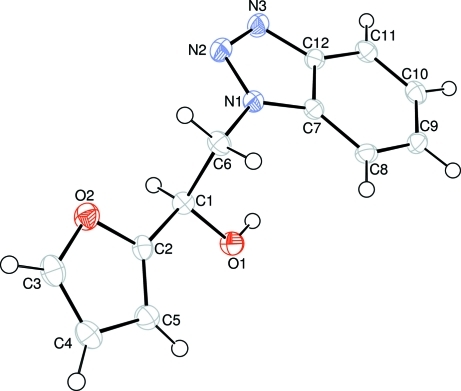
The molecular structure of the title compound with the atom-numbering scheme. Displacement ellipsoids are drawn at the 50% probability level.

**Fig. 2.**
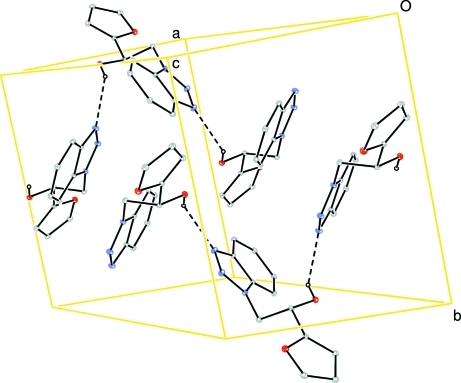
A partial packing diagram. Hydrogen bonds are shown as dashed lines. Hydrogen atoms not involved in hydrogen bonding have been omitted for clarity.

## supplementary crystallographic information

### Comment

Azole compounds have important biological activities. Benzotriazol derivatives also exhibit a good degree of analgesic, anti-inflammatory, diuretic, antiviral and antihypertensive activities (Kopanska *et al.*, 2004; Yu *et al.*, 2003; Hirokawa *et al.*, 1998). Crystal structures of similar compounds like 1-phenyl-2-(1*H*-1,2,4-triazol-1-yl)ethanol (Özel Güven *et al.*, 2008), 2-(1*H*-benzotriazol-1-yl)-1-phenylethanol (Özel Güven *et al.*, 2010), 2-(1*H*-benzotriazol-1-yl)-3-(2,6-dichlorophenyl)-1-phenylpropan-1-ol (Özel Güven *et al.*, 2011), fluconazole (Caira *et al.*, 2004), and other benzotriazole ring possesing compounds (Katritzky *et al.*, 2001; Nanjunda Swamy *et al.*, 2006) have been reported before. Now, we report herein the crystal structure of the title alcohol, (I).

In the molecule of the title compound (Fig. 1), the bond lengths and angles are generally within normal ranges. The planar benzotriazole ring [B (N1-N3/C7-C12)] is oriented with respect to the furan [A (O2/C2-C5)] ring at a dihedral angle of A/B = 24.05 (4)°. Atom C6 is 0.043 (2) Å away from the plane of the benzotriazole ring and atoms C1 and O1 are 0.010 (2) and 0.043 (1) Å away from the plane of the furan ring, respectively.

In the crystal, O—H···N hydrogen bonds (table 1) link the molecules into chains (Fig. 2). There also exist π···π contacts between the furan rings, between the triazole and benzene rings and between the benzene rings, Cg1—Cg1^i^, Cg2—Cg3^ii^ and Cg3—Cg3^ii^, may further stabilize the structure [centroid-centroid distances = 3.724 (1), 3.786 (1) and 3.8623 (9) Å; symmetry codes: (i) 1 - x, 1 - y, 1 - z; (ii) -x, 1 - y, 1 - z; Cg1, Cg2 and Cg3 are the centroids of the rings A (O2/C2-C5), C (N1-N3/C7/C12) and D (C7-C12), respectively].

### Experimental

The title compound, (I), was synthesized by reduction of 2-(1*H*-benzotriazol-1-yl)-1-(furan-2-yl)ethanone with sodiumborohydrate. A mixture of 2-(1*H*-benzotriazol-1-yl)-1-(furan-2-yl)ethanone (1010 mg, 4.44 mmol) and sodium borohydrate (561 mg, 8.89 mmol) in ethanol (50 ml) was refluxed for 4 h. After evaporation of the solvent, the mixture was neutralized with dilute HCl, and then refluxed for 30 min. After the mixture was cooled, the solution was alkalinized with dilute NaOH and the resulting precipitate was filtered. The filtrate was extracted with chloroform, then the organic phase was dried and evaporated. The residue was crystallized from 2-propanol to obtain colorless crystals suitable for X-ray analysis (yield; 634 mg, 62%).

### Refinement

H atoms were positioned geometrically with O—H = 0.82 Å (for OH group), C—H = 0.98, 0.93 and 0.97 Å for methine, aromatic and methylene H, respectively, and constrained to ride on their parent atoms, with *U*_iso_(H) = k × *U*_eq_(C,O), where k = 1.5 for OH H-atom and k = 1.2 for all other H-atoms.

### Crystal data

**Table:**

C_12_H_11_N_3_O_2_	*F*(000) = 480
*M**_r_* = 229.24	*D*_x_ = 1.384 Mg m^−^^3^
Monoclinic, *P*2_1_/*c*	Mo *K*α radiation, λ = 0.71073 Å
Hall symbol: -P 2ybc	Cell parameters from 6399 reflections
*a* = 11.3606 (4) Å	θ = 2.9–27.5°
*b* = 11.1034 (4) Å	µ = 0.10 mm^−^^1^
*c* = 8.7860 (2) Å	*T* = 120 K
β = 96.938 (2)°	Block, colorless
*V* = 1100.16 (6) Å^3^	0.50 × 0.50 × 0.20 mm
*Z* = 4	

### Data collection

**Table:**

Bruker–Nonius KappaCCD diffractometer	2531 independent reflections
Radiation source: fine-focus sealed tube	2166 reflections with *I* > 2σ(*I*)
graphite	*R*_int_ = 0.037
φ and ω scans	θ_max_ = 27.5°, θ_min_ = 3.0°
Absorption correction: multi-scan (*SADABS*; Sheldrick, 2007)	*h* = −14→14
*T*_min_ = 0.953, *T*_max_ = 0.981	*k* = −14→14
12372 measured reflections	*l* = −10→11

### Refinement

**Table:**

Refinement on *F*^2^	Secondary atom site location: difference Fourier map
Least-squares matrix: full	Hydrogen site location: inferred from neighbouring sites
*R*[*F*^2^ > 2σ(*F*^2^)] = 0.054	H-atom parameters constrained
*wR*(*F*^2^) = 0.139	*w* = 1/[σ^2^(*F*_o_^2^) + (0.0748*P*)^2^ + 0.4779*P*] where *P* = (*F*_o_^2^ + 2*F*_c_^2^)/3
*S* = 1.11	(Δ/σ)_max_ < 0.001
2531 reflections	Δρ_max_ = 0.58 e Å^−^^3^
155 parameters	Δρ_min_ = −0.55 e Å^−^^3^
0 restraints	Extinction correction: *SHELXL97* (Sheldrick, 2008), Fc^*^=kFc[1+0.001xFc^2^λ^3^/sin(2θ)]^-1/4^
Primary atom site location: structure-invariant direct methods	Extinction coefficient: 0.144 (12)

### Special details

**Table:**

Geometry. All esds (except the esd in the dihedral angle between two l.s. planes) are estimated using the full covariance matrix. The cell esds are taken into account individually in the estimation of esds in distances, angles and torsion angles; correlations between esds in cell parameters are only used when they are defined by crystal symmetry. An approximate (isotropic) treatment of cell esds is used for estimating esds involving l.s. planes.
Refinement. Refinement of F^2^ against ALL reflections. The weighted R-factor wR and goodness of fit S are based on F^2^, conventional R-factors R are based on F, with F set to zero for negative F^2^. The threshold expression of F^2^ > 2sigma(F^2^) is used only for calculating R-factors(gt) etc. and is not relevant to the choice of reflections for refinement. R-factors based on F^2^ are statistically about twice as large as those based on F, and R- factors based on ALL data will be even larger.

### Fractional atomic coordinates and isotropic or equivalent isotropic displacement parameters (Å^2^)

**Table:**

	*x*	*y*	*z*	*U*_iso_*/*U*_eq_	
O1	0.20677 (10)	0.98199 (10)	0.08354 (12)	0.0223 (3)	
H1	0.1559	0.9346	0.1048	0.033*	
O2	0.50236 (10)	1.06715 (11)	0.24705 (13)	0.0247 (3)	
N1	0.18510 (11)	0.91870 (12)	0.39959 (14)	0.0196 (3)	
N2	0.22565 (12)	0.82297 (13)	0.48488 (16)	0.0249 (3)	
N3	0.13604 (12)	0.75376 (13)	0.50674 (16)	0.0248 (3)	
C1	0.30653 (14)	0.97510 (14)	0.19621 (17)	0.0193 (3)	
H1A	0.3392	0.8933	0.1989	0.023*	
C2	0.39723 (13)	1.06237 (14)	0.15318 (17)	0.0194 (3)	
C3	0.57056 (15)	1.15175 (15)	0.18424 (19)	0.0257 (4)	
H3	0.6471	1.1732	0.2247	0.031*	
C4	0.51146 (15)	1.19920 (15)	0.05628 (19)	0.0252 (4)	
H4	0.5385	1.2581	−0.0062	0.030*	
C5	0.39773 (14)	1.14020 (15)	0.03582 (18)	0.0238 (4)	
H5	0.3367	1.1533	−0.0429	0.029*	
C6	0.26911 (14)	1.00691 (14)	0.35398 (17)	0.0214 (3)	
H6A	0.3386	1.0091	0.4298	0.026*	
H6B	0.2330	1.0862	0.3496	0.026*	
C7	0.06500 (13)	0.91156 (13)	0.36343 (16)	0.0178 (3)	
C8	−0.01985 (14)	0.98551 (14)	0.27982 (17)	0.0202 (3)	
H8	0.0010	1.0560	0.2326	0.024*	
C9	−0.13571 (14)	0.94734 (15)	0.27193 (17)	0.0226 (4)	
H9	−0.1949	0.9939	0.2182	0.027*	
C10	−0.16792 (14)	0.83958 (15)	0.34283 (17)	0.0232 (4)	
H10	−0.2474	0.8174	0.3339	0.028*	
C11	−0.08469 (14)	0.76679 (14)	0.42472 (18)	0.0222 (4)	
H11	−0.1059	0.6962	0.4713	0.027*	
C12	0.03398 (13)	0.80499 (13)	0.43403 (17)	0.0191 (3)	

### Atomic displacement parameters (Å^2^)

**Table:**

	*U*^11^	*U*^22^	*U*^33^	*U*^12^	*U*^13^	*U*^23^
O1	0.0207 (6)	0.0231 (6)	0.0226 (6)	−0.0047 (4)	0.0004 (4)	−0.0002 (4)
O2	0.0204 (6)	0.0259 (6)	0.0271 (6)	−0.0032 (4)	−0.0001 (4)	0.0002 (4)
N1	0.0181 (6)	0.0205 (6)	0.0205 (6)	0.0001 (5)	0.0030 (5)	0.0020 (5)
N2	0.0224 (7)	0.0249 (7)	0.0272 (7)	0.0045 (5)	0.0023 (5)	0.0044 (5)
N3	0.0231 (7)	0.0221 (7)	0.0293 (7)	0.0043 (5)	0.0038 (5)	0.0059 (6)
C1	0.0204 (7)	0.0172 (7)	0.0203 (7)	−0.0007 (6)	0.0028 (6)	−0.0012 (5)
C2	0.0168 (7)	0.0204 (7)	0.0213 (7)	0.0006 (6)	0.0031 (6)	−0.0038 (6)
C3	0.0203 (8)	0.0251 (8)	0.0322 (8)	−0.0052 (6)	0.0054 (6)	−0.0052 (6)
C4	0.0246 (8)	0.0237 (8)	0.0285 (8)	−0.0047 (6)	0.0087 (6)	−0.0016 (6)
C5	0.0223 (8)	0.0264 (8)	0.0228 (7)	−0.0012 (6)	0.0029 (6)	0.0007 (6)
C6	0.0201 (7)	0.0221 (8)	0.0223 (7)	−0.0039 (6)	0.0041 (6)	−0.0025 (6)
C7	0.0185 (7)	0.0178 (7)	0.0173 (7)	−0.0002 (6)	0.0034 (5)	−0.0017 (5)
C8	0.0248 (8)	0.0178 (7)	0.0184 (7)	0.0020 (6)	0.0040 (6)	0.0030 (5)
C9	0.0213 (8)	0.0274 (8)	0.0185 (7)	0.0056 (6)	0.0002 (6)	−0.0009 (6)
C10	0.0190 (7)	0.0286 (8)	0.0224 (7)	−0.0031 (6)	0.0043 (6)	−0.0042 (6)
C11	0.0243 (8)	0.0191 (8)	0.0244 (8)	−0.0022 (6)	0.0074 (6)	−0.0004 (6)
C12	0.0212 (8)	0.0169 (7)	0.0195 (7)	0.0022 (6)	0.0041 (6)	0.0004 (5)

### Geometric parameters (Å, °)

**Table:**

O1—C1	1.4139 (18)	C4—H4	0.9300
O1—H1	0.8200	C5—C4	1.440 (2)
O2—C2	1.3681 (19)	C5—H5	0.9300
O2—C3	1.375 (2)	C6—H6A	0.9700
N1—N2	1.3492 (18)	C6—H6B	0.9700
N1—C6	1.4571 (19)	C7—C12	1.401 (2)
N1—C7	1.365 (2)	C8—C7	1.404 (2)
N3—N2	1.308 (2)	C8—C9	1.376 (2)
N3—C12	1.377 (2)	C8—H8	0.9300
C1—C6	1.539 (2)	C9—H9	0.9300
C1—H1A	0.9800	C10—C9	1.417 (2)
C2—C1	1.496 (2)	C10—H10	0.9300
C2—C5	1.346 (2)	C11—C10	1.379 (2)
C3—C4	1.345 (2)	C11—C12	1.406 (2)
C3—H3	0.9300	C11—H11	0.9300
			
C1—O1—H1	109.5	N1—C6—C1	110.77 (12)
C2—O2—C3	106.13 (12)	N1—C6—H6A	109.5
N2—N1—C7	110.36 (12)	N1—C6—H6B	109.5
N2—N1—C6	119.43 (12)	C1—C6—H6A	109.5
C7—N1—C6	130.13 (13)	C1—C6—H6B	109.5
N3—N2—N1	108.96 (13)	H6A—C6—H6B	108.1
N2—N3—C12	108.40 (13)	N1—C7—C8	133.73 (14)
O1—C1—C2	107.84 (12)	N1—C7—C12	104.11 (13)
O1—C1—C6	109.45 (12)	C12—C7—C8	122.15 (14)
O1—C1—H1A	109.6	C7—C8—H8	122.0
C2—C1—C6	110.64 (12)	C9—C8—C7	115.98 (14)
C2—C1—H1A	109.6	C9—C8—H8	122.0
C6—C1—H1A	109.6	C8—C9—C10	122.27 (15)
O2—C2—C1	116.78 (13)	C8—C9—H9	118.9
C5—C2—O2	110.66 (14)	C10—C9—H9	118.9
C5—C2—C1	132.56 (14)	C9—C10—H10	119.1
O2—C3—H3	124.6	C11—C10—C9	121.81 (15)
C4—C3—O2	110.75 (14)	C11—C10—H10	119.1
C4—C3—H3	124.6	C10—C11—C12	116.47 (14)
C3—C4—C5	106.01 (14)	C10—C11—H11	121.8
C3—C4—H4	127.0	C12—C11—H11	121.8
C5—C4—H4	127.0	N3—C12—C7	108.16 (13)
C2—C5—C4	106.45 (14)	N3—C12—C11	130.53 (15)
C2—C5—H5	126.8	C7—C12—C11	121.31 (14)
C4—C5—H5	126.8		
			
C6—N1—N2—N3	−177.41 (13)	C5—C2—C1—O1	0.9 (2)
C7—N1—N2—N3	−0.44 (17)	C5—C2—C1—C6	−118.73 (19)
N2—N1—C6—C1	92.27 (16)	O2—C2—C5—C4	−0.09 (18)
C7—N1—C6—C1	−84.02 (19)	C1—C2—C5—C4	−179.58 (15)
N2—N1—C7—C8	179.68 (16)	O2—C3—C4—C5	−0.27 (18)
N2—N1—C7—C12	0.62 (16)	C2—C5—C4—C3	0.21 (18)
C6—N1—C7—C8	−3.8 (3)	N1—C7—C12—N3	−0.58 (16)
C6—N1—C7—C12	177.17 (14)	N1—C7—C12—C11	179.06 (14)
C12—N3—N2—N1	0.05 (17)	C8—C7—C12—N3	−179.77 (13)
N2—N3—C12—C7	0.34 (17)	C8—C7—C12—C11	−0.1 (2)
N2—N3—C12—C11	−179.25 (15)	C9—C8—C7—N1	−178.60 (15)
C3—O2—C2—C1	179.51 (13)	C9—C8—C7—C12	0.3 (2)
C3—O2—C2—C5	−0.07 (17)	C7—C8—C9—C10	−0.4 (2)
C2—O2—C3—C4	0.22 (18)	C11—C10—C9—C8	0.3 (2)
O1—C1—C6—N1	64.08 (16)	C10—C11—C12—N3	179.57 (15)
C2—C1—C6—N1	−177.23 (12)	C10—C11—C12—C7	0.0 (2)
O2—C2—C1—O1	−178.53 (12)	C12—C11—C10—C9	−0.1 (2)
O2—C2—C1—C6	61.80 (17)		

### Hydrogen-bond geometry (Å, °)

**Table:**

*D*—H···*A*	*D*—H	H···*A*	*D*···*A*	*D*—H···*A*
O1—H1···N3^i^	0.82	2.26	2.7968 (18)	123

Symmetry codes: (i) *x*, −*y*+3/2, *z*−1/2.
